# Integrating a Palliative Approach into Cardiogeriatric Decision-Making for Frail Older Adults with Heart Failure

**DOI:** 10.3390/geriatrics11020034

**Published:** 2026-03-25

**Authors:** Rémi Esser, Marine Larbaneix, Alejandro Mondragon, Marlène Esteban, Christine Farges, Sophie Nisse Durgeat, Marc Harboun, Olivier Maurou

**Affiliations:** 1 Cardiogeriatrics Department, Hôpital La Porte Verte, 78000 Versailles, France; marine.larbaneix@lpv.univi.fr (M.L.); alejandro.mondragon@lpv.univi.fr (A.M.); marlene.esteban@lpv.univi.fr (M.E.); christine.farges@lpv.univi.fr (C.F.); marc.harboun@lpv.univi.fr (M.H.); olivier.maurou@lpv.univi.fr (O.M.); 2 Medical Affairs, NP Medical, 33000 Bordeaux, France; sophie@satelia.eu

**Keywords:** heart failure, cardiogeriatric care, palliative care, integrated care framework, frailty, end-of-life decision-making

## Abstract

**Background**: Advanced heart failure (HF) in very old patients follows an unpredictable trajectory marked by recurrent decompensations, progressive functional decline, and high mortality. In this population, decision-making regarding goals of care and treatment proportionality is particularly complex due to multimorbidity, frailty, cognitive vulnerability, and prognostic uncertainty, and remains insufficiently addressed by conventional disease-centred heart failure pathways. **Methods**: This narrative review synthesizes current evidence from heart-failure guidelines, geriatric medicine, and palliative care literature to propose a cardiogeriatric framework for end-of-life decision-making in advanced HF. **Results**: In older adults, functional decline and geriatric vulnerability often progress independently of cardiac parameters, limiting the relevance of prognosis-based thresholds. The palliative turning point should be understood as a multidimensional process resulting from converging cardiological, geriatric, organizational, and patient-reported signals. Therapeutic decisions should be guided by proportionality between expected benefit, treatment burden, and patient priorities. Longitudinal, iterative communication is essential to align care with evolving goals. **Conclusions**: A cardiogeriatric approach integrating cardiology, geriatrics, and palliative principles supports timely palliative integration, shared decision-making, and coordinated care in very old patients with advanced HF.

## 1. Introduction

Heart failure (HF) is a chronic and progressive syndrome affecting more than 64 million people worldwide, with prevalence rising steeply with age [1,2]. In older adults, the clinical expression of HF is strongly shaped by geriatric syndromes, including frailty, functional decline, multimorbidity, and loss of autonomy, which often evolve independently of cardiac parameters and critically influence prognosis, treatment tolerance, and care priorities. Adults aged ≥80 years now represent a growing proportion of HF hospitalisations and deaths, reflecting both population ageing and improved survival at earlier stages of the disease [1,3]. Despite advances in guideline-directed medical therapy, advanced HF remains associated with high mortality, persistent symptom burden, and recurrent hospitalisations, particularly in older adults [2,4].

Recent HF guidelines have increasingly acknowledged the importance of integrating palliative principles into the management of advanced disease [1]. The 2021 European Society of Cardiology (ESC) Guidelines emphasise early palliative integration in patients with advanced HF experiencing refractory symptoms, recurrent hospitalisations, or limited tolerance to guideline-directed medical therapy, rather than restricting it to the terminal phase [1,5]. However, these recommendations remain largely disease-centred and provide limited guidance on how to operationalise palliative integration in very old patients with multimorbidity, frailty, and cognitive vulnerability [3,6]. As a result, decision-making in advanced HF is still frequently delayed, reactive, and triggered by acute deterioration rather than anticipatory planning [7,8].

In accordance with the ESC/HFA position statement, advanced HF refers to persistent severe symptoms, recurrent hospitalisations, poor quality of life despite optimal guideline-directed therapy, and limited therapeutic options [3]. In this review, “advanced HF” refers to very old adults meeting these criteria who are typically not eligible for heart transplantation or durable mechanical circulatory support, and in whom care increasingly requires proportional, goal-oriented decision-making integrating cardiology, geriatrics, and palliative principles [3,6].

Frailty has emerged as a major determinant of outcomes in HF and is now recognised as a multidimensional syndrome influencing prognosis, treatment tolerance, and quality of life [9]. Although the Heart Failure Association consensus highlighted the importance of systematic frailty assessment, evidence remains scarce in very old populations [9,10]. Patients aged ≥85 years—who account for a rapidly growing proportion of advanced HF hospitalisations and deaths—remain largely underrepresented in clinical trials and palliative care studies [11]. Recent observational and palliative HF studies indicate that, in this age group, functional decline, cognitive impairment, and loss of autonomy often drive prognosis more strongly than cardiac parameters alone [12,13,14], challenging traditional cardiology-based thresholds and underscoring the need for an integrated cardiogeriatric framework [11,15,16,17]. Despite a prognosis comparable to many advanced cancers, palliative approaches remain less frequently integrated in HF, partly because the disease course is unpredictable and punctuated by acute decompensations rather than a linear decline [5,6,7,8,18].

Accordingly, this review addresses three practical questions for clinicians caring for very old adults with advanced HF: (i) how to recognize a multidimensional palliative turning point in a non-linear trajectory; (ii) how to reassess goals of care and therapeutic proportionality as benefit–burden trade-offs evolve; and (iii) how to conduct longitudinal, uncertainty-aware communication and shared decision-making with patients and caregivers [1,3,6]. In this review, the term “palliative turning point” does not refer to a single moment in time but to a progressive and multidimensional transition in which palliative principles become increasingly central to care decisions.

According to the World Health Organization (WHO), palliative care is an approach that improves the quality of life of patients and their families facing problems associated with life-threatening illness, through the prevention and relief of suffering by means of early identification and impeccable assessment and treatment of pain and other physical, psychosocial, and spiritual problems [19]. In this article, the term “palliative approach” refers to a process-oriented model focused on proportionality, symptom burden, and alignment with patient priorities across the disease trajectory, rather than exclusively to end-of-life care. This narrative review does not aim to provide a systematic synthesis of the literature, but rather to offer a clinically oriented cardiogeriatric perspective on the palliative process in advanced HF. It focuses on three key domains: identification of the palliative turning point, therapeutic decision-making and treatment adaptation, and communication with patients and families. The added value of this review lies in reframing decision-making in advanced HF through a cardiogeriatric lens, supporting anticipatory, patient-centred decisions across cardiological, geriatric, and palliative domains. Throughout the manuscript, “geriatric vulnerability” refers to the combined burden of frailty, multimorbidity, functional decline, and cognitive impairment.

## 2. Materials and Methods

### 2.1. Study Design

This article is a narrative review aimed at providing a clinically oriented cardiogeriatric perspective on end-of-life decision-making in advanced HF. Given the conceptual and integrative nature of the topic, a narrative approach was chosen to synthesize evidence from cardiology, geriatrics, and palliative care rather than to perform a quantitative comparison of interventions. Evidence was integrated using a narrative synthesis approach, allowing conceptual integration of heterogeneous clinical, geriatric, and palliative literature.

### 2.2. Data Sources and Search Strategy

The literature was identified through targeted searches in PubMed/MEDLINE and Google Scholar, complemented by screening of key guideline and consensus documents from professional societies (including the European Society of Cardiology and the Heart Failure Association) and by manual review of the reference lists of relevant articles and reviews.

Search queries combined concepts related to HF, advanced HF, frailty, cardiogeriatrics, palliative care, and end-of-life decision-making using Boolean operators (e.g., “heart failure” AND “palliative care”; “frailty” AND “heart failure”; “advanced heart failure” AND “decision making”).

### 2.3. Search Period and Keywords

The search focused primarily on articles published between January 2015 and September 2025 while allowing inclusion of earlier seminal studies where necessary to support key conceptual developments. Key search terms included “heart failure,” “advanced heart failure,” “frailty,” “cardiogeriatrics,” “older adults”, “elderly”, “geriatrics”, “palliative care,” “end-of-life decision-making,” and “integrated care”.

### 2.4. Methodological Considerations

No formal systematic review, meta-analysis, or risk-of-bias assessment was performed, as the objective was not to quantify treatment effects but to integrate heterogeneous clinical, geriatric, and palliative evidence into a coherent conceptual framework. Accordingly, no formal quality appraisal of included studies was undertaken, as the aim was to synthesize conceptual and practice-oriented insights rather than to provide an exhaustive or quantitative comparison of interventions. This approach reflects the current lack of randomized data specifically addressing end-of-life decision-making in very old patients with advanced HF and aligns with the exploratory and hypothesis-generating intent of this review.

Given the narrative and clinically oriented objective of this review, references were selected to illustrate key concepts rather than to provide an exhaustive coverage of the rapidly expanding advanced HF literature. The aim was not to replicate existing guideline- or device-focused reviews, but to integrate cardiology, geriatrics, and palliative care perspectives in a population underrepresented in advanced HF trials.

## 3. Understanding the Trajectory of Advanced HF in Older Adults

### 3.1. Disease Trajectory and Prognostic Uncertainty

HF typically follows a fluctuating trajectory, with periods of relative stability interrupted by acute decompensations requiring hospitalisation [7,20]. Each episode of decompensation is associated with increased mortality risk, loss of functional capacity, and diminished quality of life [3,21]. Unlike oncology, however, HF lacks a clearly identifiable final stage of life, making prognostic discussions particularly challenging [22].

Several prognostic models have been developed, including the Seattle Heart Failure Model and MAGGIC score [22,23]. While useful at a population level, their discriminative performance is limited in very old patients with multimorbidity and frailty [24]. As a result, clinicians often underestimate mortality risk and overestimate survival, contributing to delayed end-of-life planning [24].

Importantly, poorer outcomes observed in older patients with HF should not be attributed to chronological age alone. Growing evidence suggests that frailty and physiological vulnerability, rather than age per se, are the primary drivers of prognosis and treatment tolerance. Failure to distinguish frailty from chronological age carries a risk of inappropriate therapeutic limitation and age-based decision-making. Objective assessment of frailty and functional reserve is therefore essential to avoid ageism and to ensure that disease-modifying therapies are withheld only when disproportionate to physiological vulnerability rather than age [25].

### 3.2. Advanced HF in the Elderly

In line with the ESC/HFA position statement, advanced HF refers to patients with persistent severe symptoms, recurrent hospitalisations, and poor quality of life despite optimal guideline-directed medical therapy, often associated with end-organ dysfunction and limited therapeutic options. In very old patients, these criteria are frequently met in the absence of eligibility for heart transplantation or durable mechanical circulatory support, placing clinical decision-making at the interface between advanced HF management and palliative-oriented care [3].

Advanced HF in older adults differs substantially from that observed in younger populations. Older patients are rarely eligible for advanced therapies such as heart transplantation or left ventricular assist devices and instead experience progressive congestion, renal dysfunction, sarcopenia, and declining physiological reserve [3,16]. In this context, functional decline and loss of autonomy often outweigh cardiac parameters alone in shaping prognosis and care needs [11,18].

**Figure 1:**
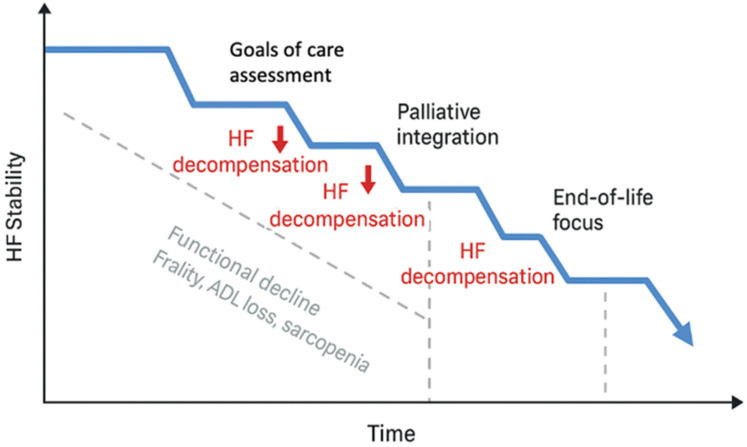
Trajectory of advanced heart failure in older adults. HF progression is characterized by a non-linear course with recurrent acute decompensations superimposed on a gradual decline in functional reserve. In older adults, functional deterioration and frailty often progress continuously, independently of cardiac events. Repeated points of reassessment and integration of palliative care are therefore required along the trajectory rather than being confined to the final months of life.

From a cardiogeriatric perspective, advanced HF in older adults should therefore be understood as a multidimensional condition resulting from the interaction between cardiac dysfunction, geriatric vulnerability—understood here as the multidimensional interaction between frailty, multimorbidity, functional decline, and cognitive impairment—and social factors, rather than as a purely cardiac end-stage disease [17]. The complex and non-linear trajectory of advanced HF in older adults is summarized in Figure 1.

## 4. Identifying the Palliative Turning Point in HF

In this review, the concept of a “palliative turning point” is not intended to define a discrete stage or abrupt transition. Rather, it refers to a progressive and often iterative process through which palliative principles become increasingly central as disease-modifying options lose proportionality. This approach is fully compatible with contemporary HF models, in which palliative care is introduced early and evolves alongside active treatment. Disease-modifying and palliative approaches frequently coexist until very late stages of HF, requiring continuous reassessment rather than a binary shift in care objectives [5,7].

### 4.1. Cardiological Markers of Transition

Several clinical features may signal progression toward a palliative phase of HF, including recurrent hospitalisations for decompensation, persistent New York Heart Association (NYHA) class III–IV symptoms despite optimized therapy, refractory congestion, hypotension limiting treatment escalation, and progressive right-sided failure or pulmonary hypertension [3,4,26,27]. Intolerance to guideline-directed therapies due to renal dysfunction or symptomatic hypotension further reflects reduced physiological reserve [1,28].

### 4.2. Geriatric Markers of Vulnerability

Geriatric indicators are crucial for identifying patients approaching a palliative phase. Frailty, commonly assessed using phenotypic or clinical frailty scales, is highly prevalent in advanced HF and independently associated with mortality, hospitalisation, and poor quality of life [11,16,18]. However, frailty assessment should not rely on a single instrument. Functional performance measures, such as walking speed or simple gait tests, as well as cognitive assessment, provide complementary and clinically meaningful information in older HF patients.

Decline in activities of daily living, recurrent falls, weight loss, malnutrition, and cognitive impairment often precede terminal events and strongly influence patient experience and care priorities [16,17,18]. These markers reflect global vulnerability rather than cardiac disease severity alone and support a transition toward symptom-focused, goal-oriented care.

In addition to clinical indicators, organisational and system-level factors may signal the need for earlier palliative integration, particularly when fragmented care pathways lead to repeated crisis-driven hospitalisations or poor coordination between care settings [13].

Taken together, cardiological, geriatric, organisational, and patient- and caregiver-related signals supporting the identification of a palliative turning point in advanced HF are summarised in Table 1.

In clinical practice, identification of this transition relies on the convergence of multiple domains rather than a single trigger, typically combining recurrent decompensations, functional decline, frailty progression, and increasing treatment intolerance.

### 4.3. Prognostic Tools and Their Limitations

Although prognostic scores may help frame discussions, they cannot substitute for comprehensive clinical judgment in older patients with HF [22,23,24]. Their discriminative performance is limited in the presence of multimorbidity, frailty, and cognitive impairment, which are common in very old populations. Given the inherent uncertainty and non-linear trajectory of HF, cardiogeriatric practice therefore emphasizes early and iterative discussions about prognosis and goals of care rather than waiting for prognostic certainty [9,24].

Importantly, assessment of vulnerability in older adults with HF should not rely on a single instrument. Objective functional measures such as gait speed have been consistently associated with mortality and hospitalisation in older adults with heart failure [10,16]. While frailty scales such as the Fried phenotype or the Clinical Frailty Scale provide a global estimate of vulnerability, objective functional measures are essential to capture real-life physiological reserve. Simple walking tests, including gait speed or short-distance walk tests, provide robust prognostic information and are strongly associated with mortality, hospitalisation, and loss of autonomy in older HF patients.

Cognitive assessment is equally crucial, as cognitive impairment is frequent in advanced HF and directly influences treatment adherence, decision-making capacity, and communication regarding goals of care. Integrating frailty scales with functional performance measures and cognitive evaluation allows a more comprehensive and clinically meaningful cardiogeriatric assessment, better suited to guide anticipatory decision-making in advanced HF.

## 5. Therapeutic Decision-Making in Advanced HF: A Cardiogeriatric Framework

In contemporary HF care, advanced disease is typically managed within specialized referral pathways, including evaluation for heart transplantation, durable left ventricular assist devices (as bridge or destination therapy), or chronic inotropic support. These strategies represent the standard life-prolonging options for selected patients with advanced HF [3,11]. However, a substantial proportion of very old patients with advanced HF are not eligible for such interventions due to age, frailty, comorbidity burden, or patient preferences. In these situations, the central clinical dilemma is not whether advanced therapies exist, but when they are no longer proportionate or appropriate, and how to transition toward care focused on symptom relief and quality of life.

### 5.1. Reassessing Goals of Care

Therapeutic decision-making in advanced HF should begin with clarification of patient goals and values. Studies consistently show that many older patients prioritize symptom relief, functional preservation, and avoidance of hospitalisation over life prolongation [29,30,31,32]. These preferences may change over time and require regular reassessment.

In cardiogeriatrics, therapeutic decision-making is guided less by disease stage alone than by the principle of therapeutic proportionality, defined as the balance between expected clinical benefit, treatment burden, and patient priorities. In practice, this assessment involves weighing expected survival benefit and time-to-benefit against treatment burden (including polypharmacy, adverse effects, and care complexity), together with functional reserve and the patient’s stated priorities. In very old patients with advanced HF, this balance is frequently altered by reduced physiological reserve, multimorbidity, and functional or cognitive vulnerability.

While cardiology guidelines primarily focus on disease severity and treatment escalation, a cardiogeriatric approach emphasizes whether a given intervention remains proportionate to the patient’s overall condition, goals of care, and capacity to tolerate treatment. Therapeutic proportionality therefore shifts the clinical question from “Can this treatment be prescribed?” to “Should this treatment be pursued in this specific patient at this stage of their trajectory?”.

Therapeutic proportionality is particularly relevant in the context of multimorbidity, where competing priorities and treatment-related trade-offs are common, especially in advanced HF when chronic kidney disease, cognitive impairment, or frailty limit physiological reserve. For example, aggressive diuretic or neurohormonal intensification may improve congestion at the expense of renal function, orthostatic symptoms, or cognitive clarity, potentially undermining the patient’s functional autonomy and quality of life.

A cardiogeriatric framework explicitly acknowledges these conflicts and supports individualized prioritization of outcomes, favoring symptom relief, functional preservation, and care coherence over maximal disease-centered optimization when proportionality is no longer achieved.

### 5.2. Treatment Adaptation and De-Escalation

As HF progresses, the balance between benefit and harm of certain therapies may shift. A cardiogeriatric approach supports individualized treatment adaptation, including deprescribing when medications no longer align with patient goals or increase adverse effects [26,27,28,33]. Randomized evidence suggests that discontinuation of preventive medications in patients with limited life expectancy does not worsen outcomes and may improve quality of life [33].

Decisions regarding implantable cardioverter-defibrillators are particularly complex. ICD shocks near the end of life are common and distressing, yet discussions about device deactivation are frequently delayed [29,34]. Anticipatory, patient-centered conversations are therefore essential.

In selected older patients with recurrent HF decompensations, the use of long-term venous access devices may facilitate intermittent intravenous diuretic administration at home, potentially reducing hospitalisations related to congestion management [30,35]. When feasible and supported by adequate caregiver or home-care resources, such approaches may improve symptom control and quality of life. However, their use should be carefully weighed against infection risk, treatment burden, and overall goals of care [30,35].

### 5.3. Integrating Palliative Care

Multiple studies and randomized trials demonstrate that early integration of palliative care in HF improves symptom burden, quality of life, and patient satisfaction without increasing mortality [7,8,9]. Palliative care should thus be viewed as complementary to cardiogeriatric management rather than as an alternative or terminal option.

## 6. Communication with Patients and Families: The Cardiogeriatric Approach

### 6.1. Challenges in HF Communication

Communication in advanced HF is complicated by prognostic uncertainty, fluctuating disease course, and emotional distress [24,29]. Unlike many oncological trajectories, HF often lacks clearly identifiable final months of life and follows a non-linear course, which complicates the timing and framing of communication about future care. Cognitive impairment, common in elderly HF patients, further complicates discussions and necessitates adaptation of communication strategies [17,18]. Assessment of cognitive status and basic functional mobility should therefore be considered upstream of communication, as they directly influence patients’ understanding, decision-making capacity, and the feasibility of shared discussions with families.

### 6.2. Principles of Effective Communication

Effective communication relies on early, honest, and repeated conversations that are tailored to cognitive and emotional capacities [31,32]. Rather than focusing solely on life expectancy, discussions should address anticipated symptoms, care preferences, and acceptable trade-offs between longevity and quality of life.

### 6.3. The Cardiogeriatric Communication Mindset

Communication in advanced HF requires more than the transmission of medical information. In cardiogeriatrics, it is conceived as a progressive and iterative process, adapted to fluctuating clinical status, cognitive vulnerability, and emotional readiness. Rather than a single end-of-life discussion, cardiogeriatric communication relies on temporal sequencing, repetition, and reformulation to ensure shared understanding. Studies on serious illness communication have emphasized the importance of iterative conversations acknowledging uncertainty in chronic illness trajectories [31,36,37].

A central component of this mindset is the translation of complex medical uncertainty into meaningful, patient-centered terms while explicitly acknowledging prognostic ambiguity. Accepting and communicating uncertainty—rather than avoiding it—allows patients and families to engage more realistically with evolving goals of care and supports trust in the therapeutic relationship [32].

### 6.4. Shared Decision-Making

Shared decision-making integrates clinical expertise with patient values and preferences and is central to ethical end-of-life care [36,37]. Advance care planning, when conducted proactively, improves concordance between delivered care and patient wishes and reduces unnecessary interventions near the end of life [36].

## 7. Ethical and Organisational Considerations

Ethical challenges in advanced HF include avoiding non-beneficial interventions, respecting patient autonomy, and addressing decisional capacity in cognitively impaired patients [26,29]. Organizational barriers such as fragmented care pathways and limited access to palliative services further complicate care delivery [11,38,39,40].

From a geriatric care perspective, decision-making in advanced HF is inseparable from organisational and system-level factors. Very old patients frequently transition between hospital, outpatient care, nursing facilities, and home settings, making coordination between hospital-based teams, primary care physicians, home-care services, and nursing facilities essential. Inadequate care coordination contributes to fragmented trajectories, repeated emergency admissions, and delayed goal-of-care discussions. Caregiver burden is also a critical but often overlooked determinant of care feasibility, influencing treatment adherence, place-of-care decisions, and the sustainability of home-based management. An integrated cardiogeriatric framework that ensures continuity of care across settings, including structured communication with nursing facilities and caregivers, is therefore central to anticipatory and proportionate decision-making in advanced HF.

**Figure 2:**
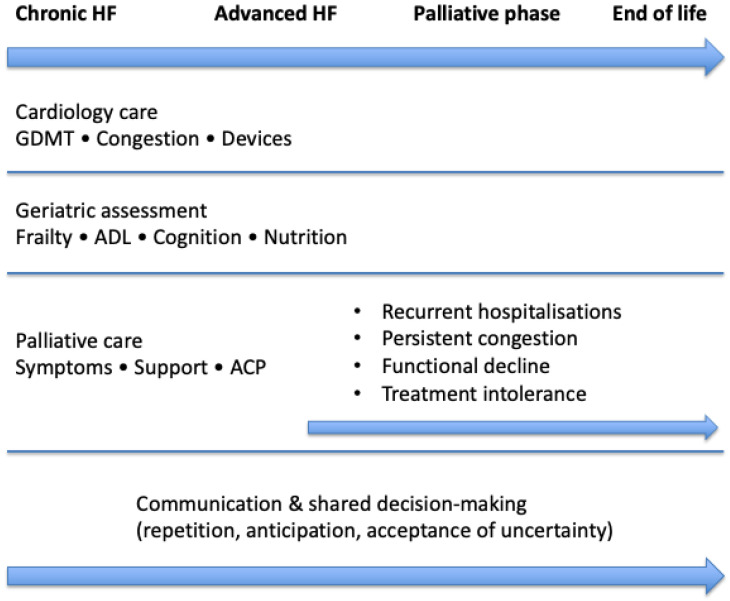
Integrated cardiogeriatric and palliative care framework in advanced heart failure. Rather than a late and abrupt transition, cardiology care, geriatric assessment, and palliative care are progressively combined along the disease trajectory. Repeated communication and shared decision-making support anticipatory planning, therapeutic proportionality, and continuity of care across settings.

These observations support the development of an integrated cardiogeriatric framework that progressively combines cardiology, geriatrics, and palliative principles across care settings, rather than relying on isolated end-of-life interventions. This framework aims to strengthen continuity, anticipatory planning, and coherence of care. An example of such an integrated framework is illustrated in Figure 2.

In advanced HF, conflicts between healthcare teams, patients, and families often arise from misaligned expectations rather than from disagreement about facts. A cardiogeriatric communication mindset—based on early, repeated, and transparent discussions—may help prevent such conflicts by progressively preparing all stakeholders for therapeutic transitions. By explicitly addressing uncertainty and revisiting goals of care over time, this approach reduces the risk of crisis-driven decisions and supports shared understanding when treatment limitations become necessary.

Evidence supports the value of an integrated cardiogeriatric framework linking cardiology, geriatrics, and palliative principles across care settings, which has been associated with improved outcomes and more appropriate healthcare utilization [11,38,39,40,41].

## 8. Future Perspectives and Unmet Needs

Future priorities include earlier identification of palliative needs, improved integration of geriatric assessment into HF care pathways, and greater inclusion of very old patients in clinical research [1,16,41]. Patients aged 85 years or older remain largely underrepresented in HF trials, despite accounting for a growing proportion of those with advanced disease. Future studies should therefore focus on outcomes that matter most to this population, including symptom burden, functional status, quality of life, and place of care, rather than survival alone.

Beyond pharmacological interventions, there is a critical need to evaluate integrated cardiogeriatric care models—particularly coordinated cardiology, geriatrics, and palliative pathways—using patient-centered and system-level outcomes. Such research may help improve care coherence, reduce non-beneficial interventions, and better align treatments with patient goals at the end of life. These priorities also have important implications for health policy and healthcare system design, supporting the development of care models adapted to the needs of an ageing HF population.

Several studies have highlighted persistent unmet needs in end-of-life care for patients with advanced HF, particularly regarding anticipatory communication, symptom control, and care coordination, which remain insufficiently addressed in routine practice, especially in very old and frail patients [13].

## 9. Limitations

This narrative review has limitations, including the absence of a systematic or quantitative synthesis and reliance on clinical expertise, which limits causal inference. Nevertheless, it offers practical guidance to support clinical decision-making, promote earlier palliative integration, and structure an integrated cardiogeriatric care framework bridging cardiology, geriatrics, and palliative care. In addition, the proposed framework may be influenced by heterogeneity in geriatric practices and international variability in healthcare organisation and care frameworks, which may limit direct generalisability across settings. In addition, as in most narrative reviews, the selection of references may be subject to selection bias due to the absence of a systematic selection process and formal quality appraisal.

## 10. Conclusions

In ageing heart failure (HF) populations, therapeutic efficacy alone is insufficient to guide clinical decision-making. Frailty, multimorbidity, and functional vulnerability increasingly shape prognosis and patient experience, requiring a shift toward person-centred goals and proportional care.

This narrative review proposes a cardiogeriatric framework for very old adults with advanced HF—patients frequently underrepresented in trials and often ineligible for transplant- or device-based pathways. Within this framework, therapeutic proportionality is defined as the balance between expected benefit, treatment burden, and patient priorities, and should be reassessed iteratively as functional reserve and competing risks evolve across the HF trajectory.

Rather than a late transition from “active treatment” to “palliation”, cardiogeriatric care integrates cardiology, geriatrics, and palliative principles progressively, supported by longitudinal communication and shared decision-making under prognostic uncertainty. This approach offers a pragmatic and ethically grounded response to the growing complexity of cardiovascular care in ageing societies and supports future research on relevance-driven, integrated models of care in frail older adults with HF.

## Figures and Tables

**Table 1:** Signals Suggesting a Palliative Turning Point in Advanced Heart Failure.

Domain	Signals Suggesting a Palliative Turning Point
Cardiological factors	Recurrent hospitalisations for HF decompensation Persistent congestion despite optimized therapy Progressive right-sided failure or pulmonary hypertension Hypotension or renal dysfunction limiting guideline-directed medical therapy Intolerance or diminishing response to HF treatments
Geriatric factors	Decline in activities of daily living (ADL/iADL) Progressive frailty or sarcopenia Unintentional weight loss or malnutrition Cognitive decline or recurrent delirium Recurrent falls or loss of mobility
Organisational factors	Increasing healthcare utilization (ED visits, unplanned admissions) Recurrent need for urgent treatment escalation Fragmented care or poor coordination between settings Difficulty maintaining outpatient follow-up Transition to institutional care (EHPAD, nursing facility)
Patient- and caregiver-related factors	Patient-reported symptom burden despite treatment Expressed fatigue with repeated hospitalisations Loss of treatment meaning or motivation Caregiver exhaustion or inability to cope Explicit questions about prognosis or end-of-life issues

Rather than defining a strict threshold, the palliative turning point in advanced HF should be understood as the convergence of multiple cardiological, geriatric, organizational, and subjective signals. This multidimensional assessment supports anticipatory goal redefinition and timely integration of palliative approaches. These domains are not hierarchical and should not be interpreted as a scoring system. Rather, they are context-dependent signals whose cumulative presence may support clinical recognition of a palliative transition.

## Data Availability

The datasets used and/or analysed during the current study are available from the corresponding author (R.E.) on reasonable request. Due to patient privacy restrictions, data are not publicly available.
